# Development of Potential Yeast Protein Extracts for Red Wine Clarification and Stabilization

**DOI:** 10.3389/fmicb.2019.02310

**Published:** 2019-10-09

**Authors:** Leonor M. Gaspar, Amadeu Machado, Rute Coutinho, Susana Sousa, Raquel Santos, Adriana Xavier, Manuel Figueiredo, Maria de Fátima Teixeira, Filipe Centeno, João Simões

**Affiliations:** ^1^Genomics Unit, Biocant, Cantanhede, Portugal; ^2^PROENOL – Indústria Biotecnológica, Lda., Canelas, Portugal

**Keywords:** fining agents, vinification, turbidity, color, wine

## Abstract

Recently, new technologies have been combined to improve quality and sensorial diversity of wine. Several fining agents were developed to induce flocculation and sedimentation of particulate matter in wine, enhancing its clarification, and stabilization. The fining agents most commonly used are animal proteins, such as milk casein or egg albumin. However, its use is being related to food intolerance. To overcome this issue, alternative sources should be explored for use in industrial processes. In previous studies performed by our consortium, the potential of yeast protein extracts (YPE) in white wine clarification, stabilization, and curative processes was identified. Thus, the main objective of the present work is to select YPE with the potential to develop fining agents for red wine, without health risk to consumers. Therefore, five yeast strains were selected from a diversified collection of oenological yeasts, in order to produce protein extracts. Along with the fining trials, a vinification assay was performed to evaluate the maceration effect of the obtained YPE. The previously selected yeast strains were also screened for the production of the usual enzymatic activities found in commercial maceration preparations, namely polygalacturonase, cellulase, protease, and ß-glucosidase activities, in order to evaluate its potential effect on wine. Our results indicate that YPE, particularly BCVII 1, BCVII 2, and BCVII 5 were able to promote a significant brilliance increase, along with a turbidity reduction and final color improvement. In the vinification assay, BCVII 2 stands out with better results for color intensity and phenolic compounds content improvement. In what refers to enzymatic activities, BCVII 2 shows advantage over the other YPEs, due to its protease and β-glucosidase activity. We demonstrate that the selected YPEs, with emphasis on BCVII 2, may represent an efficient alternative to the commonly used fining products.

## Introduction

The high competitiveness in the winemaking industry leads to a constant need to innovate and optimize products and techniques in order to produce wines with high-quality standards that satisfy the demands of an increasingly attentive and selective consumer (Siezen, 2008; Fernandes et al., 2015).

Fining agents are traditionally applied during wine production to obtain a brighter and clarified product as a result of the elimination of particles responsible for turbidity (Armada and Falqué, 2007). These agents are also beneficial to soften tannic intensity, enhance mouthfeel perception and to improve wine filterability, and stabilization (Tschiersch et al., 2010; Mierczynska-Vasilev and Smith, 2015; Mekoue Nguela et al., 2016).

This process also reduces the protein instability, which is a great improvement (Cosme et al., 2012), because the presence of unstable proteins in wines is currently a great concern for winemakers (Batista et al., 2009). In this process is expected the elimination of suspended particles and certain compounds that are responsible for oxidation reactions and undesirable astringency of wine, enhancing its organoleptic characteristics and improving wine’s visual aspect (Castillo-Sánchez et al., 2008; Lochbühler et al., 2015). In this case, wine color, brightness, and limpidity are crucial sensory attributes that are greatly valued by the consumers, and may cause a wine to be accepted or rejected (González-Neves et al., 2014). According to the type of wine and the desired effect, it is necessary to choose the suitable fining agent to be applied (Tschiersch et al., 2010).

The fining agents that are most commonly used in the wine industry can be obtained from animal proteins, vegetable proteins, and inorganic compounds (Chagas et al., 2012). These proteins reveal a wide diversity of molecular masses, isoelectric points, and/or surface charge densities, and depending on the conditions of each wine, they will interact differently with specific wine components (Versari et al., 1998; Cosme et al., 2008; Tschiersch et al., 2010).

Protein fining agents have the ability to form complexes with wine tannins, creating negatively charged hydrophobic colloids. In the presence of metal cations, these colloids become insoluble and tend to precipitate, after flocculation (Chagas et al., 2012; Mierczynska-Vasilev and Smith, 2015). Contrarily, proteins that do not interact with tannins have the tendency to combine with particles in suspension or in colloidal solution, which most of them are negatively charged, leading as well to its sedimentation (Chagas et al., 2012). Other proteins as caseins are insoluble at the low wine pH, and so they tend to coagulate and flocculate. However, to induce its precipitation and wine clarification, it is required the presence of tannins (Ribéreau-Gayon et al., 2006; Chagas et al., 2012). Simultaneously, they are capable to bind and consequently eliminate phenolic compounds that affect the color and taste of wines (Weber et al., 2007).

Though these fining agents seem to have a high efficacy to wine stabilization and clarification, some of these may endanger the consumer’s health (Monaci et al., 2013). For instance, milk casein and ovalbumin from hen’s egg white, both from animal sources, were described as allergenic substances and may cause food intolerances (Monaci et al., 2013). This type of fining agents belongs to the “hidden allergens,” which are allergenic ingredients present in complex foods and cannot be recognized by the common consumer, and so they are not predicted (Weber et al., 2007; Deckwart et al., 2014). This fact brings up various concerns for the average consumer, since there is the risk of food intolerances and allergic reactions, leading consumers to be more thoughtful about what they consume. Since these issues may put the consumer’s health at risk, the European Union implemented a regulation (Regulation EU No 1169/2011) that demands the labeling of all substances and products that may cause these allergies and intolerances. Posteriorly, Regulation (EC) No. 579/2012 was also implemented in order to obligate the labeling of all fining treatments based on milk, milk-based or egg, and egg-based products that are used in grape must and wine. The implementation of these regulations is extremely important since they give all the information to the consumer, allowing a conscious consumer choice (Holzhauser et al., 2003; Deckwart et al., 2014; Fernandes et al., 2015).

Alternatively, non-protein fining agents are currently being used in wines, including polyvinylpolypyrrolidone (PVPP), bentonite, activated charcoal, and chitosan (Chagas et al., 2012; Mierczynska-Vasilev and Smith, 2015). PVPP acts similarly to proteinaceous fining agents and exhibits highly selective adsorption of phenolic substances, specifically to anthocyanins and catechins, reducing their amount in wine (Mierczynska-Vasilev and Smith, 2015; Gil et al., 2017). For instance, polyphenols are essential components of red wines, however, when in excess, they may cause undesirable features, such as bitterness and astringency (Hufnagel and Hofmann, 2008; Margalit, 2015). Despite not being usually applied in red wines, PVPP improves wine color and brightness, reducing also its bitterness, without stripping the wine’s aroma (Gil et al., 2017; Martínez-Lapuente et al., 2017). Regarding bentonite, it is a cation exchanger clay, which carries a negative net charge and so, interacts electrostatically with the positively charged proteins, at wine pH (Lambri et al., 2010; Vincenzi et al., 2015; Jaeckels et al., 2017). When added to the wine, it removes unstable proteins through precipitation (Margalit, 2015). However, bentonite action seems to be non-specific for proteins (Waters et al., 1992), as it also removes other charged species or aggregates that may be beneficial molecules for wine aroma and color (Pretorius, 2000; Lambri et al., 2010). This contributes to the loss of sensory quality that is often claimed in wines treated with bentonite (Vincenzi et al., 2015), furthermore, treatments with this fining agent can mean a large loss of the wine volume, from 5% to 20%, as bentonite lees (Lagace and Bisson, 1990). Additionally, bentonite raises a number of environmental issues, since it is associated with occupational health risks, including dust inhalation, and disposal of hazardous bentonite waste (Van Sluyter et al., 2015).

Due to health issues related with animal protein agents and the high environmental impacts from bentonite and PVPP, the need to find a different approach based on substances that do not endanger the consumer’s health has become a challenge. To overcome this concern, yeast protein extracts (YPE), obtained from wine and grape endogenous yeasts seem to be a potential alternative. In fact, several studies show that the application of novel fining treatments based on yeast derivatives improved the quality of the wine, by decreasing its turbidity and astringency (Dupin et al., 2000; Caridi, 2006; Iturmendi et al., 2010).

Yeast protein extracts can be obtained from distinct components of yeasts, including the cytoplasm, vacuole, or the cell wall (Lochbühler et al., 2015) and are applied during winemaking process with different purposes (Pozo-Bayón et al., 2009). For instance, in several yeast species, including Saccharomyces cerevisiae, the protein extraction occurs from the cell wall, because it is composed by an external layer of highly glycosylated mannoproteins covalently linked to an amorphous matrix of β-glucans from the inner layer of the wall (Lipke and Ovalle, 1998; Klis et al., 2002; Lochbühler et al., 2015). However, mannoproteins show lower binding affinity to tannins of red wine when compared with non-glycosylated proteins (Lochbühler et al., 2015). Therefore, because the presence of polysaccharides and mannoproteins may inhibit the precipitation of proteins of the fining agent with wine compounds, particularly tannins (De Freitas et al., 2003; Rowe et al., 2010) it is crucial the development of YPEs derived specifically from the soluble extract after the total degradation of the cytoplasmic content and the elimination of the cell walls. Depending on the production method they can be categorized into distinct types: inactive yeasts (obtained by thermal inactivation and drying of the yeasts), yeast autolysates (thermal inactivation followed by an incubation step allowing enzymatic activities and cell wall degradation), yeast hulls or walls (yeast walls without cytoplasmic content), and yeast extracts (the soluble extract of the cytoplasmic content, after elimination of the cell walls) (Pozo-Bayón et al., 2009).

Yeast protein extracts are not classified as food allergens by European laws and, since 2013, are allowed for fining of grape must and wine within the European Union (Regulation EC No 144/2013). The appliance of YPEs as fining agents is also allowed by International Organization of vine and wine (OIV) [resolutions OIV-OENO 416-2011 and OENO 417-2011] and established a monograph [resolution OIV-OENO 452-2012] offering a new alternative to the wine sector.

Another traditional practice in winemaking industry is the addition of maceration enzymes to improve the clarification and filtration of wine, the release of varietal aromas from precursor compounds, which enhances the wine flavor and aroma, and the reduction in alcohol levels (Charoenchai et al., 1997; Van Rensburg and Pretorius, 2000). Commercial maceration preparations are complex enzymatic mixtures, mainly pectinases, but also cellulases, hemicellulases, and proteases (Pardo et al., 1999; Fernández et al., 2000; Romero-Cascales et al., 2007; Merín et al., 2015). These enzymes are also produced and secreted by oenological yeasts and could have a beneficial effect in wine production.

The aim of this study is to evaluate the potential applicability of protein extracts, obtained from wine endogenous yeasts, as an effective substitute/alternative to the traditional fining agents in the enhancement of red wine clarification and stabilization. To reach this objective, several oenological aspects were assessed including chromatic analysis, turbidity, and lees production. We also explore the maceration effect of the YPE, through a microvinification assay, and screening the main enzymatic activities of yeasts that make up the commercial preparations developed for that purpose.

## Materials and Methods

### Wines and Fining Agents

For this experiment, a red wine of 2017 from a local producer was received after alcoholic fermentation was completed but still turbid and unstable. Until used, it was stored protected from light at a temperature of 22 ± 2°C, and the sulfur dioxide was rectified to avoid their premature evolution. Four fining products (Albumin, Bentonite, Casein, and Vegetable Protein derived from hydrolyzed wheat gluten) were used as a reference for the fining experiments and prepared according to the supplier recommendation. Red wine of 2017 and fining agents were kindly provided by Proenol (Canelas, Portugal).

### Yeast Protein Extracts (YPE)

According to preliminary fining trials, high biomass production, and total protein content, five specific yeast strains, isolated from spontaneous wine fermentation, were selected from a diverse collection of oenological yeasts in order to evaluate their potential as fining agents in red wines. The selected yeast strains were used to produced YPE through a confidential methodology developed and optimized at laboratory scale (Fernandes et al., 2015). Thus, the selected yeast species and strains originated the corresponding YPE: *Pichia anomala* (BCVII 1), *Metschnikowia pulcherrima* (BCVII 2), *Saccharomyces bayanus* (BCVII 3), *Lachancea thermotolerans* (BCVII 4), and *Saccharomyces cerevisiae* (BCVII 5).

### Fining Experiments

#### Protein Fining Agents Characterization

Total protein content was accessed by Pierce^TM^ BCA Protein Assay Kit (Thermo Fisher Scientific, United States) according to the kit manufacturer’s protocol, using bovine serum albumin as the standard. In agreement with OIV resolution [Resolution OIV-OENO 452-2012], the protein molecular weight profile of each protein fining agent was acquired by sodium dodecyl sulphate-polyacrylamide gel electrophoresis (SDS-PAGE). Protein samples were prepared in Laemmli Sample Buffer [100 mM Tris–HCl (pH 6.8), 4% (w/v) SDS, 0.01% bromophenol blue (w/v), 0.2% glycerol (v/v), 0.02% β-mercaptoethanol (v/v)]. Proteins were denatured by boiling samples for 5 min at 99°C. SDS-PAGE were performed according to the method of Laemmli (1970). Samples were loaded on 4% stacking gel and run on 12.5% resolving acrylamide gel containing 10% SDS. Gel electrophoresis was performed on a Bio-Rad Protean II apparatus with power supply set at 80 V/gel for the stacking gel and 140 V/gel for the resolving gel. Gels were then stained Coomassie Blue R250 reagent.

#### Fining Trials

After receiving the untreated red wine, the reference fining agents (x4) and the YPE (x5) were tested simultaneously. Fining assays were conducted in triplicates, during 48 h, using glass tubes of 110 mL of total volume. Just before the fining trials, each tube was filled in with 95 mL of wine and the experiments were conducted at a controlled temperature of 22 ± 2°C, and protected from light. The fining agents were applied in wine according to the supplier recommendations and under minimum and maximum dosages defined by The Australian Wine Research Institute (AWRI) and OIV. The applied maximum dosage for YPE was determined according to OIV regulation (OENO 417- 2011), which states that the maximum dose to be used must not exceed 60 g/hL for red wines. The tested dosages are presented in Table 1. Fining products were left to flocculate and sediment to the bottom of the tubes for 48 h. Clarified wine samples (supernatants) were then carefully pipetted and filtered through a qualitative paper filter, pore size 20 μm, to new tubes. Wine samples were posteriorly analyzed by the following wine analytical methodologies.

**TABLE 1:** Fining agents respective dosages applied in the trials.

**Fining products**	**Minimum dosage (g/hL)**	**Maximum dosage (g/hL)**
Albumin	5	15
Bentonite	20	50
Casein	5	25
Vegetable Protein	10	80
BCVII 1	20	60
BCVII 2	20	60
BCVII 3	20	60
BCVII 4	20	60
BCVII 5	20	60

### Wine Analysis

#### Conventional Oenological Parameters

Free and total SO_2_ was controlled using SO_2_ – Matic 23 Crison (Hach Lange, Spain). pH and conductivity were controlled using an HI 5522 (Hanna Instruments, Rhode Island, United States).

Turbidity was measured at 540 nm, with a BioTek PowerWave XS (BioTek, Vermont, United States) using a 96-well plate, under a pathlength of 0.2 cm.

#### Lees Volume

Lees volume was obtained by measuring the thickness of the sediment in the glass tubes after the fining trials were completed (Fernandes et al., 2015). Results were expressed as a percentage of the initial volume of wine (% v/v).

#### Chromatic Characteristics

Each wine sample was evaluated by measuring absorbance at 450, 420, 570, and 630 nm in a 96-well plate under a pathlength of 0.2 cm, using BioTek PowerWave XS (BioTek, Vermont, United States). The colorimetric characteristics are defined by the coordinates clarity (L^∗^) (L^∗^ = 0 black and L^∗^ = 100 colorless), red/green color component (a^∗^) (a^∗^ > 0 red, a^∗^ < 0 green), blue/yellow color component (b^∗^) (b^∗^ > 0 yellow, b^∗^ < 0 blue), and its derived magnitudes chroma (C^∗^) and tone (H^∗^). The coordinates were calculated using the CIELab system in the MSCV^®^ 7 software, from Grupo de Color de La Rioja (Logroño, Spain) (Pérez-Magariño and González-San José, 2002).

#### Wine Unstable Proteins

To precipitate the wine unstable proteins, KDS method was performed (Vincenzi et al., 2005). A 10% stock solution of sodium-dodecyl sulfate (SDS, Sigma) was prepared and then added to a 1 mL wine sample, in order to achieve final concentrations of 0.1%. Samples were gently mixed for 2 min and then heated up to 95°C for 5 min. Potassium chloride 2 M (KCL, Sigma) was added to each sample for a final concentration of 200 mM. Samples were set to incubate for 30 min in a rotating mixer, and protein pellets were recovered after centrifugation at 14,000 g for 15 min at 4°C. Phosphate buffered saline (1×) pH 7.4 (PBS buffer) was then added to each sample in order to dissolve the pellets.

After protein precipitation, protein content present in wine samples was measured by Pierce^TM^ BCA Protein Assay Kit (Thermo Fisher Scientific, United States) and the protein molecular weight pattern of each wine was assessed by SDS-PAGE electrophoresis.

### Maceration Effect

#### Vinification Assay

A laboratory scale vinification was performed to evaluate the macerative effect of our YPEs in the enhancement of red wine final color. For this assay, berries of *Vitis vinifera* Baga grape variety from Anadia region, Portugal, were destemmed, crushed, and squeezed. The resultant grape must was separated from the solid phase (grape skins and seeds) and the free SO_2_ level was corrected to 15 mg/L, with potassium metabisulfite, to prevent spontaneous fermentation by native yeasts present in the grapes.

Alcoholic fermentations were carried out by the must inoculation with selected pure *S. cerevisiae* commercial yeast, according to manufacturer instructions. Individual fermentations were carried out in 50 mL tubes containing 22 g of must. Finally, the equivalent weight of grape skins and seeds, correspondent to the 22 g of grape juice, was added to each the tube for maceration, in an approach similar to industrial scale process. In order to study the maceration effect our yeast extracts, minimum, and maximum dosages allowed by OIV for YPE (OIV-Oeno 494-2012) were added to distinct tubes. Besides the 5 YPE, 4 commercial fining agents tested in this work and a commercial maceration preparation (CMP) were used. Individual fermentations were controlled by the loss of weight along the time, measured twice a day, up to decrease rate stabilizes. Until the end of this process, the cap, formed by the grape skins and seeds, was punched down once a day. These tests were performed in triplicate, at room temperature. The maceration effects on the post-fermentation wine were evaluated through wine color intensity (CI) and total POLYPHENOL Content (TPC).

#### Color Intensity

CI was determined spectrophotometrically and calculated as the sum of the absorbance at 420, 520, and 620 nm (Glories, 1984).

#### Total Phenolic Content

The total phenolic content was measured spectrophotometrically at 750 nm through The Folin–Ciocalteu index (FCI) using a standard curve of gallic acid (0–1 g/L), and the results were expressed as mg of gallic acid equivalents per liter (mg GAE/L) (Singleton and Rossi, 1965).

### Enzymatic Characterization of Yeast

The extracellular production of polygalacturonase, protease, ß-glucosidase, and cellulase enzyme activities was screened for the yeast strains: *P. anomala* (BCVII 1), *M. pulcherrima* (BCVII 2), *S. bayanus* (BCVII 3), *L. thermotolerans* (BCVII 4), and *S. cerevisiae* (BCVII 5). Tests were carried out on plates with agar media containing differential substrates suitable for each particular activity. All yeasts isolates were, previously, grown on YPD plates (containing 1% yeast extract, 2% peptone, 2% glucose, and 2% agar) and then inoculated in YPD liquid medium at a final concentration of 10^4^ cells/mL, at 30°C. After the yeasts have reached their exponential state, cell counting was performed using TC10^TM^ Automated Cell Counter (BioRad Laboratories, California, United States). Then approximately 150 cells of each yeast strain were plated, in triplicate, on the mediums described below to test for extracellular enzyme activity.

#### Polygalacturonase Activity

Polygalacturonase production was screened plating the yeasts onto polygalacturonate agar medium containing 1.25% polygalacturonic acid (Sigma), 0.68% potassium phosphate, 0.67% yeast nitrogen base, 1% glucose, and 2% agar, with adjusted pH of the medium to 3.5. The plates were incubated for 5 days, at 30°C. The colonies were rinsed off the plates with distilled water before staining the plates with 0.1% ruthenium red. A purple halo around the colonies indicates positive activity.

#### Protease Activity

Yeasts were screened for protease production on YPD plates containing 2% skim milk powder (Sigma). The plates were incubated for 5 days at 30°C. A clear zone around the colony identified protease activity (Strauss et al., 2001).

#### ß-Glucosidase Activity

Based on the work of Arévalo Villena et al. (2005), ß-Glucosidase activity was evaluated by plating the yeasts into selective medium containing 2% yeast extract, 1% peptone, 1% cellobiose (4-O- ß-D-glucopyranosyl-D-glucose, Sigma), and 2% agar. The pH of the media was adjusted to 5 before autoclaving. Before pouring into the plates, 2 mL of a filter-sterilized 1% ammonium ferric citrate solution was added to 100 mL media. The plates were incubated for 5 days at 30°C. Colonies with activity were identified by discoloration of the media to a brown color (Strauss et al., 2001).

#### Cellulase Activity

Cellulase production was assessed by plating the yeast onto YPGE plates (containing 1% yeast extract, 2% peptone, 3% glycerol and 2% ethanol) with 0.4% carboxymethylcellulose (CMC, Sigma), as described by Belda et al. (2016). The plates were incubated for 5 days at 30°C. The colonies were flooded with 0.1% Coomassie Brilliant Blue R 250 and were incubated with for 5 to 7 days and then washed with distilled water, according to Gohel et al. (2014).

### Statistical Analysis

Statistical analysis was performed using GraphPad Prism software version 6.0, for windows. Means and standard deviations values were calculated, one way Analysis of Variance (ANOVA) tests was performed and significant differences were evaluated by the Bonferroni’s multiple comparisons test. Results were considered significant at *p* < 0.05.

## Results and Discussion

### Protein Fining Agents

**FIGURE 1:**
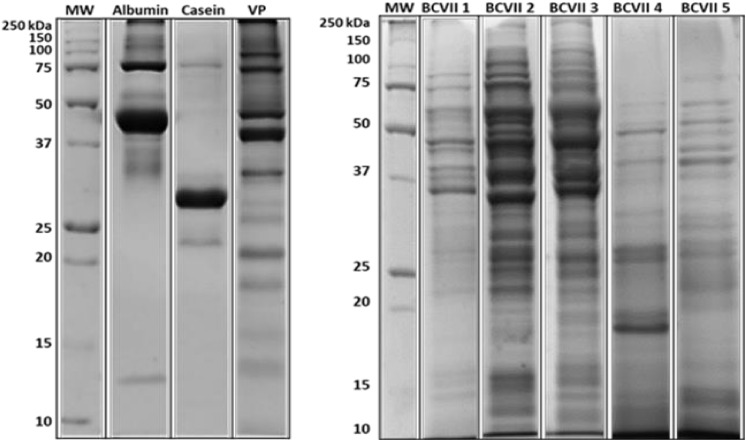
Protein molecular weight profile. Protein samples of the fining agents tested in this study by Coomassie-stained SDS-PAGE. BioRad Precision Plus Protein^TM^ as the protein weight standard. VP, vegetable protein.

In terms of protein composition, SDS-PAGE electrophoresis (Figure 1) showed that at least 50% of the total yeast protein is above 15 kDa of molecular weight, which is in accordance with the OIV demand (OIV-Oeno 494-2012).

Similarly to the reported by Cosme et al. (2007) and Gambuti et al. (2012), albumin appeared as the largest band on the gel, showed at ≈ 43 kDa, which corresponds probably to albumin isoform 2 and 3. The other presented bands are expected to be the remaining isoforms. In the case of casein, it is a heteroprotein which contains four principal phosphoproteins and phosphoglycoproteins (α_*S*__1_-casein, α_*S*__2_-casein, κ-casein, and β-casein). Through the analysis of the acrylamide gel (Figure 1 and Supplementary Figures S1, S2), several bands are located at ≈ 24, 30, and 34 kDa, which probably, respectively, correspond to α_*S*__1_-casein, α_*S*__2_-casein and β-casein subunits (Ribéreau-Gayon et al., 2006; Cosme et al., 2007). Regarding the vegetable protein (VP), it was observed a great distribution of different protein bands from 10 to 100 kDa, which is according to the expected, once these bands correspond to distinct glutenin subunits (Chinuki et al., 2013; Fernandes et al., 2015).

In the case of bentonite, its protein profile was not evaluated as it is not from protein origin. When comparing the protein profile of the commercial fining products with the YPEs, it is shown a greater distribution of the protein bands between the molecular weight ranges of 10–150 kDa, in the case of YPEs. This information suggests that YPEs may have a wider interaction with the wine unstable proteins.

### Clarification Potential

#### Lees Volume

**FIGURE 2:**
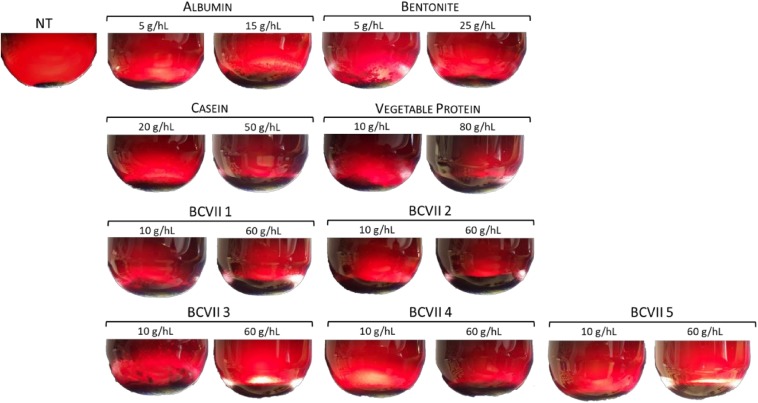
The aspect of lees after 48 h of treatment by the application of the referenced fining products and the selected yeast protein extracts. NT, not treated wine.

After 48 h of fining treatment, lees production was perfectly visible in all conditions (Figure 2). The reference fining products showed a high lees volume variation (Table 2), displaying values from 0.175%, in the case of bentonite maximum dosage (25 g/hL), to 2.300% in VP minimum dosage (10 g/hL). In what refers to casein, it presented values of 0.467 and 0.848% at the minimum and maximum dosage, respectively. These high values were due to the fact that caseins are insoluble at the low wine pH, and so they tend to coagulate and flocculate.

**TABLE 2:** Lee volume percentage values obtained after fining trials with the different fining agents and YPE.

**Lee volume (% v/v) Mean ± SD**		

**Fining products**	**Min. dosage**	**Max. dosage**
Albumin	0.293 ± 0.082	0.791 ± 0.092
Bentonite	0.350 ± 0.050	0.175 ± 0.025
Casein	0.467 ± 0.094	0.848 ± 0.034
Vegetable protein	2.300 ± 0.000	2.150 ± 0.450
BCVII 1	0.450 ± 0.150	0.818 ± 0.083
BCVII 2	0.775 ± 0.035	1.400 ± 0.000
BCVII 3	0.567 ± 0.125	0.775 ± 0.035
BCVII 4	0.500 ± 0.141	0.433 ± 0.047
BCVII 5	0.359 ± 0.116	0.609 ± 0.092

*Mean and standard deviation values were calculated concerning the three replicates.*

In the case of YPEs, the volume percentage and homogeneity were found to be similar to those obtained with albumin and casein application. However, the lees were found to be more compact and homogeneous. This evidence is in agreement with previous results obtained in our laboratory by Fernandes et al. (2015) for white wine. Fining trials with red wine were described by Iturmendi et al. (2010) and Lochbühler et al. (2015) with gelatin as reference agent, and both of the investigation groups reported a reduction in lees production in wine treated with YPEs revealing a fining improvement, since the decrease of lees means a higher yield in wine.

The final aspect and thickness of the lees produced by YPEs showed great potential as an alternative clarification agent, since they may contribute for better wine filtration and also reduction of wine loss on the bottom of the vat. Also, unlike bentonite, YPEs have no constraints in terms of process handling or dregs disposal, as they are harmless and biodegradable (Fernandes et al., 2015).

#### Final Turbidity

**FIGURE 3:**
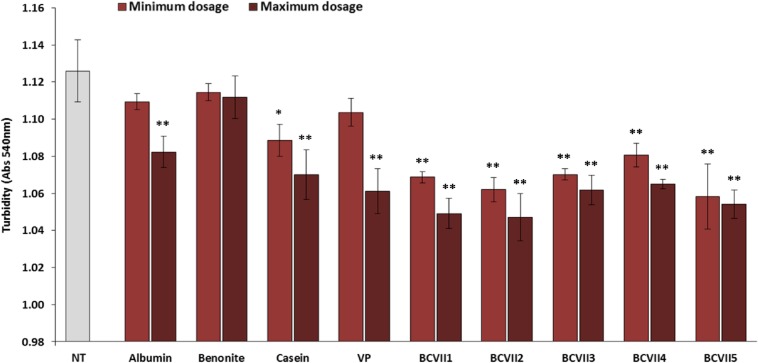
Final turbidity of wine after 48 h of fining trials. NT, non-treated wine; VP, vegetable protein; BCVII 1 to BCVII 5, treated wine samples. Bars indicate mean ± SD (*n* = 3), ^∗^ denote significant differences at *p* < 0.05, ^∗∗^ denote significant differences at *p* < 0.01.

After the fining tests, the impact of the YPE and reference fining agents, on wine final turbidity was compared. The results revealed that YPE exhibit a better capability to reduce wine turbidity (Figure 3). According to the work of Fernandes et al. (2015) this tendency was already been verified in white wines. In what refers to reference fining agents, bentonite showed the lowest turbidity reduction both at the minimum and maximum dosages. This evidence may be due to the fact that bentonite is a cation exchanger clay and so, it interacts only with the positively charged proteins. On the other hand, albumin, casein and vegetable protein showed a better capacity for turbidity reduction, at the maximum dosage, when comparing to bentonite. When the YPEs maximum dosage (20 g/hL) was applied, the turbidity decreased significantly, showing better results with BCVII 1, BCVII 2 and BCVII 5. This evidence shows that YPEs have, in fact, a behavior similar to the reference fining agents, with a superior capacity for turbidity reduction.

#### Color Characterization

**FIGURE 4:**
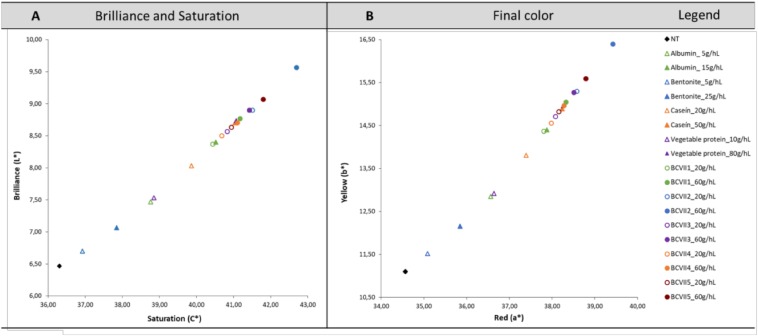
Chromatic characterization using the CIELab system. **(A)** Saturation (C^∗^) and Brilliance (L^∗^). **(B)** Red (a^∗^) and Yellow (b^∗^) values. Results were obtained before and after treatment of red wine with the reference fining products and YPE.

CIELab method was used in order to determine the chromatic characteristics of the untreated and treated wine. Figure 4A reveals that in all treated wines, the brilliance (L^∗^) increased significantly, which is in agreement with other studies performed in red wine fining, namely Cosme et al. (2007) and González-Neves et al. (2014). These results were more prominently observed when the wine was treated with YPE, most notably with BCVII 2, BCVII 3, and BCVII 5. Along with this increase, it was also verified an increase in the saturation (C^∗^), resulting in a wine with a more intense color (Figure 4B). Regarding the wine color after the fining trails (Figure 4B), it was observed a color improvement in the wine when treated with YPE. When compared with the reference fining products, wines treated with YPE, most notably BCVII 2, BCVII 3 and BCVII 5, were found to be more reddish (a^∗^) and simultaneously more yellowish (b^∗^). These tendencies reveal that YPE has a superior capacity to promote a significant brilliance increase, and final color improvement, which is in agreement with the results obtained in terms of turbidity reduction.

### Maceration Effect

**FIGURE 5:**
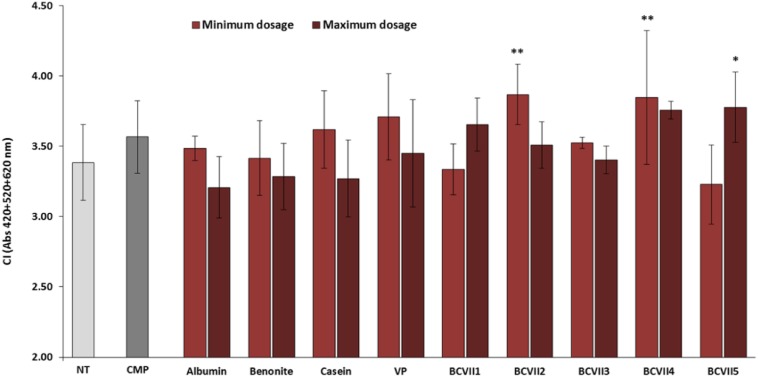
Color intensity (CI) of wine after the vinification assay. NT, non-treated wine; CMP, commercial maceration preparation; VP, vegetable protein; BCVII 1 to BCVII 5, treated wine samples. Bars indicate mean ± SD (*n* = 3), ^∗^ denote significant differences at *p* < 0.05, ^∗∗^ denote significant differences at *p* < 0.01.

**FIGURE 6:**
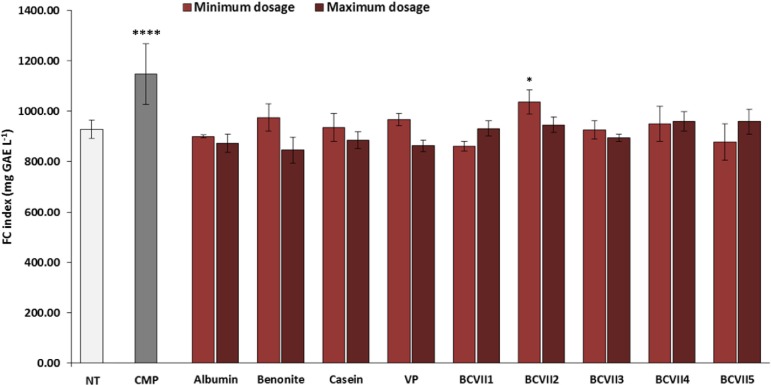
Phenolic compounds index of wines (Folin-Ciocâlteu method) after the vinification assay. NT, non-treated wine; CMP, commercial maceration preparation; VP, vegetable protein; BCVII 1 to BCVII 5, treated wine samples. Bars indicate mean ± SD (*n* = 3), ^∗^ denote significant differences at *p* < 0.05, ^*⁣*⁣**^ denote significant differences at *p* < 0.0001.

The macerative effect of the YPEs in the investigation was evaluated, after a laboratory scale vinification, in relation to wine final color and phenolic compound extraction. The addition to the wine of maceration enzymes that hydrolyze pectin, hemicellulose, and cellulose, structural polysaccharides that provide firm structure to the grapes, is a common practice in winemaking process for the enhancement of red wine final color (Romero-Cascales et al., 2007; Merín et al., 2015). Among the YPEs investigated, 3 of them (BCVII 2, BCVII 4, and BCVII 5) significantly improved wine CI, in comparison with the non-treated wine (Figure 5). Regarding total polyphenol content (Figure 6), only when BCVII 2, in minimum dosage, was added to the wine, significant differences were observed. In this particular assessment, CMP appeared highlighted as a best enhancer of the polyphenol content being BCVII 2 the YPE closest to this result. Besides the contribution to the color enhancement, higher extraction of phenolic compounds led to an increased formation of polymeric pigments resulting in better stability in aged wines (Escribano et al., 2017). Analyzing the previous aspects together, BCVII 2 stands out as the unique yeast strain capable of improve, in a significate way, the color intensity and phenolic compound extraction. Addition of pectolytic enzymes was reported to produce red wines with significantly higher values of CI, AC, and other phenolic compounds (Kelebek et al., 2009). Similar results were found by Romero-Cascales et al. (2007) in an investigation with different commercial macerating enzymes.

Among the reference fining agents, also studied for maceration effect, just the lowest dosage of casein presented a significant variation in one of the assayed components. None of them promoted significant alterations on TPC and CI.

The exhibited ability by YPEs to increase the values of the analyzed parameters could makes them a viable option to apply also as a color and phenolic enhancer in red wine production, since high colored wines, with good structure and roundness, are a final product increasingly sought by the consumer (Romero-Cascales et al., 2007).

### Yeast Screening of Enzymatic Properties

Since we are studying the oenological applicability of oenological yeast extracts with a wide spectrum of distinct proteins, the yeasts strains selected to produce the YPEs were also tested for the presence of several enzymatic activities, with potential interest in winemaking. Pectinases, cellulases, glycosidases, and proteases are enzymes produced by wine yeasts that may give an important benefit during the wine production (Ducruet et al., 1997; Pardo et al., 1999; Fernández et al., 2000; Romero-Cascales et al., 2007). These enzymatic activities were screened, on specific agar medium plates, due to their influence on certain properties, as turbidity and filterability of wine (Belda et al., 2016).

#### Polygalacturonase Activity

In oenological yeasts, the presence of polygalacturonase activity, which is the most commonly encountered pectic enzymes, is observed with low frequency. This is due to the fact that most *S. cerevisiae* strains do not exhibit positive activity (Merín et al., 2011). Among 78 yeast isolates Merín et al. (2013), 9 tested positive for enzymatic activity, with just a single isolate belonging to *S. cerevisiae* species. For non-*Saccharomyces* yeasts, Charoenchai et al. (1997) screened twenty-six strains and the activity was not found in any of the tested yeasts, Strauss et al. (2001) had just 9 positive results among 245 yeast isolates, better results were reported by Fernández et al. (2000) who had identified polygalacturonic activity in 45% of a total of 182 yeast isolates. About our screening tests, none of the strains exhibited polygalacturonase activity. The absence of this activity is indicative that yeast strains would not have a big influence on the pectin composition of the juice and wine treated with the selected strains.

#### Cellulase Activity

**FIGURE 7:**
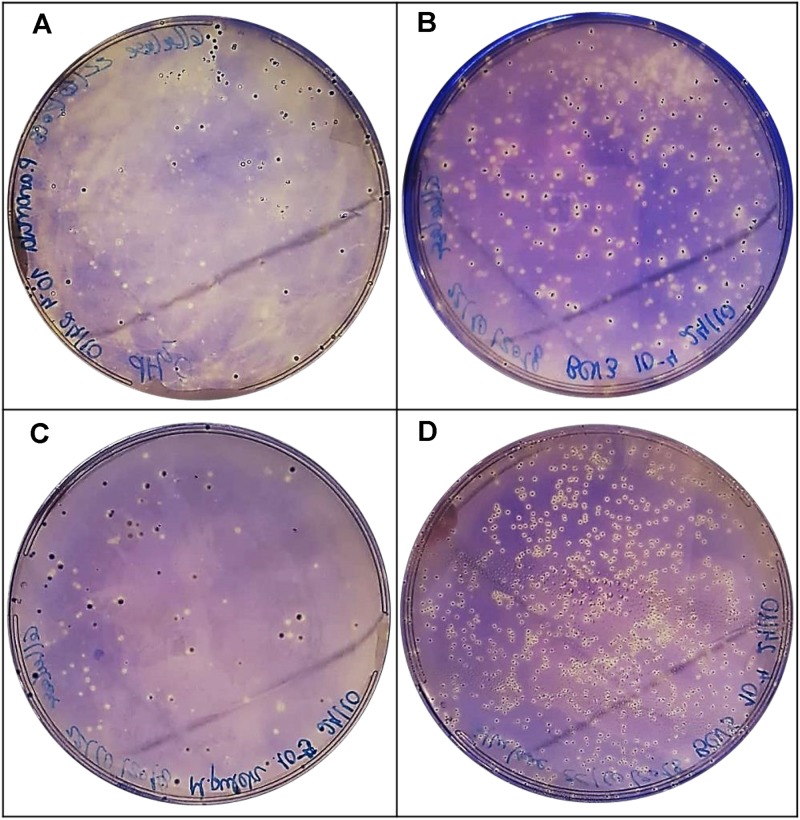
Positive results for cellulase production. Zone of clearance in **(A)** *P. anomala* BCVII 1. **(B)** *M. pulcherrima* BCVII 2, **(C)** *L. thermotolerans* BCVII 4. **(D)** *S. cerevisiae* BCVII5 on agar plate after incubation at 30°C and Coomassie Brilliant Blue R-250 coloration.

Cellulase activity is involved in the improvement of skin maceration and color extraction of grapes, as well as in the quality, stability, and filtration of wines (Escribano et al., 2017). This evidence is observed since cellulose, a carbohydrolase the same way as polygalacturonase, may degrade pectin in grape cell walls (Claus and Mojsov, 2018). In this case, *P. anomala* BCVII 1, *M. pulcherrima* BCVII 2, *L. thermotolerans* BCVII 4 and *S. cerevisiae* BCVII 5 showed a moderate cellulase production, leaving out only *S. bayanus* BCVII 3 (Figure 7 and Table 3). This production was not as evident as protease or β-glucosidase, however, a clear zone was observed around each colony of the 4 yeasts. Cellulase activity was previously identified in non-*Saccharomyces* yeast strains and described by Strauss et al. (2001) and Escribano et al. (2017). The same way as polygalacturonase, cellulase activity improves the release of reducing sugars which may be an improvement on the aromatic profile of wines (Strauss et al., 2001; Romero-Cascales et al., 2007).

**TABLE 3:** Production of protease, β-glucosidase, and cellulase by the selected wine yeast strains.

**Yeast strain**	**Protease**	**β-glucosidase**	**Cellulase**
*P. anomala* BCVII 1	−	−	++
*M. pulcherrima* BCVII 2	+++	+++	++
*S. bayanus* BCVII 3	−	−	+
*L. thermotolerans* BCVII 4	−	−	++
*S. cerevisiae* BCVII 5	−	−	++

*−, no growth; +, weak production; ++, moderate production; +++, intense production.*

#### Protease Activity

**FIGURE 8:**
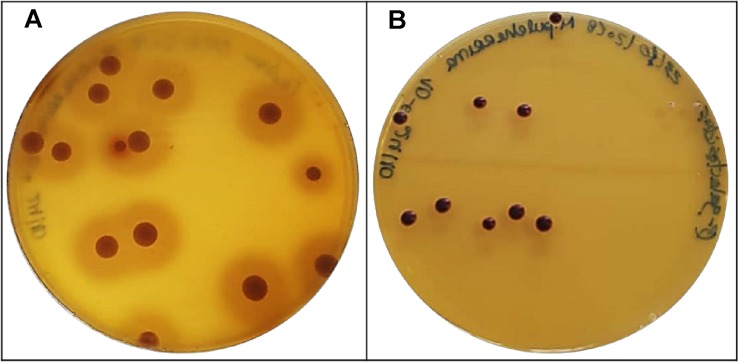
Positive results for protease and β-glucosidase activity in *M. pulcherrima* BCVII 2. **(A)** Zone of clearance showing protease production on agar plate after incubation at 30°C. **(B)** Brown colonies showing β-glucosidase activity on agar plate after incubation at 30°C.

Proteases are extremely relevant in winemaking since they can accelerate autolysis process and degradation of grape proteins responsible for wine protein instability. Therefore, proteases may improve wine stabilization by preventing protein haze, and most importantly reducing bentonite demand (Claus and Mojsov, 2018). Protein hydrolysis into smaller peptides and amino acids is an effective approach to increase clarification and stabilization of must and wines (Fernández et al., 2000). Thus, all 5 yeasts were analyzed for this activity, but only *M. pulcherrima* BCVII 2 presented a distinguished protease production (Table 3) identified by an intense zone of clearance in each colony (Figure 8A). Proteolytic activity was screened by Strauss et al. (2001) and Charoenchai et al. (1997) in wine yeasts. Both working groups found enzymatic activity in several non-*Saccharomyces* isolates. This evidence reveals that *M. pulcherrima* BCVII 2 has a greater potential to be used in winemaking, not only due to the impact on protein haze occurrence but also to the extraction of the phenolic compounds from the grape cell membrane which protease catalytic activity help to disrupt (Romero-Cascales et al., 2007).

#### β-Glucosidase Activity

Volatile odorous compounds, present in free form in grapes, enhance the flavor of must and wine. These volatile compounds, however, can bind to sugar molecules and form odorless non-volatile glycosidic complexes (Gunata et al., 1988). β-Glucosidases cleave these sugar-conjugated compounds releasing the aroma precursors to their free form, improving the wine aroma (Fernández et al., 2000). Besides this desired feature, β-glucosidases exhibit the ability to degrade anthocyanins into other molecules, which may result in a decrease of wine color (Ducruet et al., 1997; Romero-Cascales et al., 2007). β-glucosidase activity was found in Saccharomyces cerevisiae and described in several investigations (Delcroix et al., 1994; Rosi et al., 1994; Hernández et al., 2003; Arévalo Villena et al., 2005) and Non−*Saccharomyces* yeasts (Rosi et al., 1994; Arévalo Villena et al., 2005; Cordero Otero et al., 2006). In our experiments for β-glucosidase activity screening, *M. pulcherrima* BCVII 2 stands out in relation to the other 4 yeasts (Table 3), showing a significant production (Figure 8B), perceived by a discoloration of each colony to a brown color. Also, yeasts that may produce β-glucosidases are resistant to ethanol and low pH, which is an advantage over the remaining yeasts (Claus and Mojsov, 2018).

## Conclusion

In the present work, we demonstrate the great potential of YPEs in red wine clarification and stabilization as an alternative to the fining agents most commonly used in the wine industry. The selected YPE, particularly BCVII 1, BCVII 2, and BCVII 5, were able to promote brilliance increase, along with a turbidity reduction and final color improvement, when compared with the reference fining agents.

The appliance of these alternative fining agents during maceration of red wine also seems to be a valid option, since they enhanced the phenol compound extraction and final color intensity, where BCVII 2 appear as the better YPE in the conducted experiments. In what refers to enzymatic activities, *M. pulcherrima* BCVII 2 stands out again in relation to the other YPEs, due to its protease, and β-glucosidase activity. This information indicates the need to determine the impact that these activities may have on the sensory properties of wine.

Hence, we believe that this work will be an important contribution to the development of a new biological product which will improve the final quality of red wines. The selected YPEs, with emphasis on BCVII 2, represent an efficient alternative to the commonly used fining products, without compromising the wine final quality or the consumers’ health. Furthermore, the yeast strains selected and studied in this investigation could also be a potential source for the commercial production of enzymes with biotechnological interest for use in the wine industry.

## Data Availability Statement

The datasets generated for this study are available on request to the corresponding author.

## Author Contributions

LG, AM, RC, and JS designed the experiments. LG, AM, RC, SS, RS, AX, and MF performed the experiments. MT, FC, and JS conceived the project and edited the manuscript. LG and AM analyzed the results and wrote the manuscript.

## Conflict of Interest

AX, MF, MT, and FC were employed by company PROENOL. The remaining authors declare that the research was conducted in the absence of any commercial or financial relationships that could be construed as a potential conflict of interest.
