# Supplementary material for: Development of Potential Yeast Protein Extracts for Red Wine Clarification and Stabilization
Source: Front Microbiol. 2019 Oct 9;10:2310. doi: 10.3389/fmicb.2019.02310 (PMC6794431; doi:10.3389/fmicb.2019.02310)
Supplement: Supplementary file 1 [file Data_Sheet_1.PDF]

# *Supplementary Material*

## 1.1 Supplementary Figures

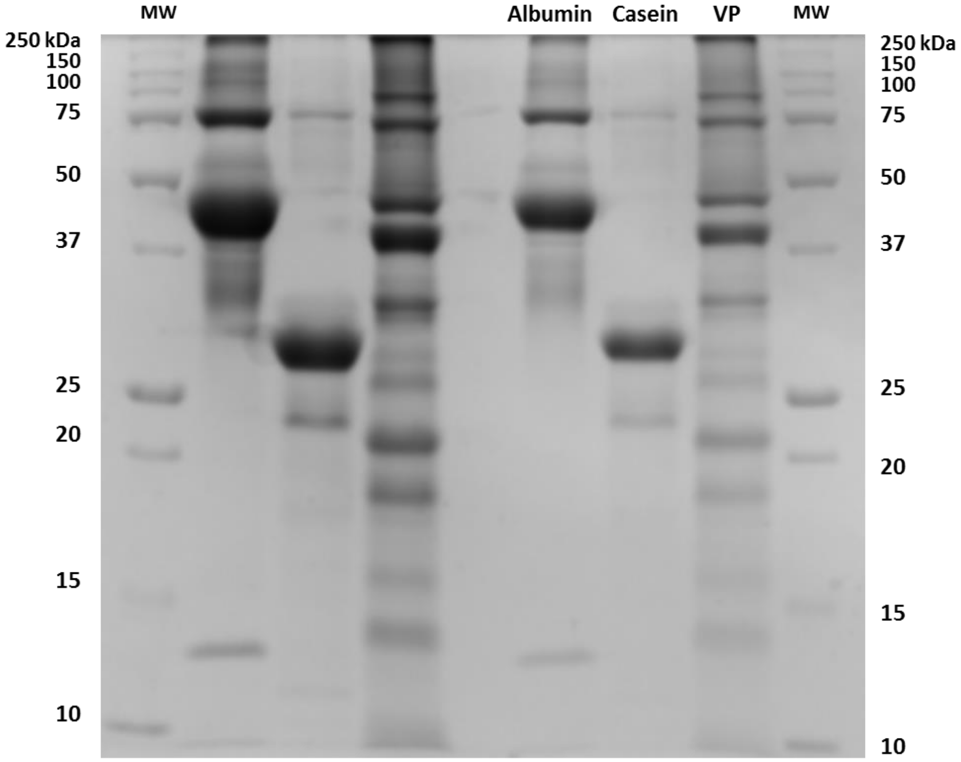


**Supplementary Figure 1.** Original Gel image from Figure 1. Protein molecular weight profile. Protein samples of the fining agents tested in this study by Coomassie-stained SDS-PAGE. BioRad Precision Plus Protein™ as the protein weight standard. VP, vegetable protein.

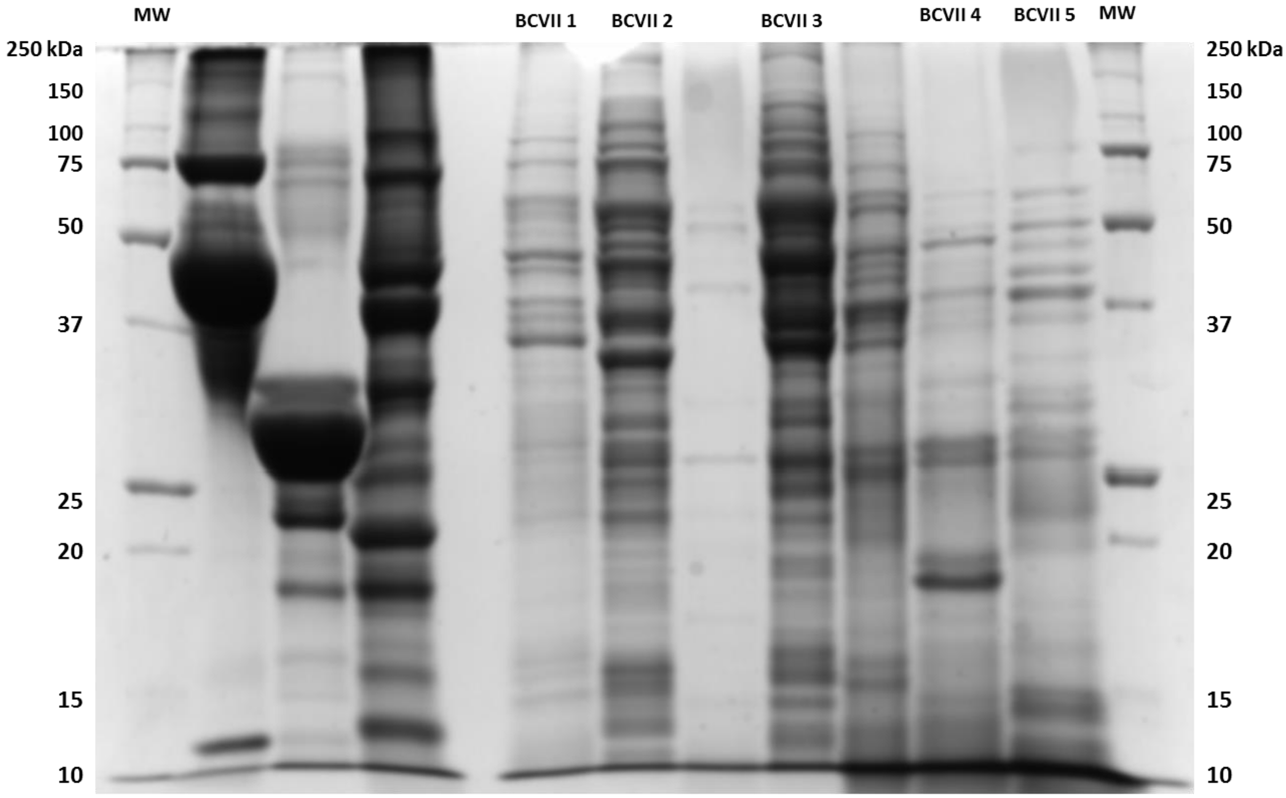


**Supplementary Figure 2.** Original Gel image from Figure 1. Protein molecular weight profile. Protein samples of the fining agents tested in this study by Coomassie-stained SDS-PAGE. BioRad Precision Plus Protein™ as the protein weight standard.
